# 非小细胞肺癌中EMT的发生机制及预后价值的研究进展

**DOI:** 10.3779/j.issn.1009-3419.2014.07.13

**Published:** 2014-07-20

**Authors:** 勤琛 曹, 路军 赵, 平 王

**Affiliations:** 300060 天津，天津医科大学肿瘤医院放疗科，国家肿瘤临床医学研究中心，天津市“肿瘤防治”重点实验室 Department of Radiotherapy, Tianjin Medical University Cancer Institute and Hospital, National Clinical Research Center of Cancer, Key Laboratory of Cancer Prevention and Therapy, Tianjin 300060, China

**Keywords:** 肺肿瘤, 上皮细胞-间叶细胞转化, 机制, 预后, Lung neoplasms, Epithelial to mesenchymal transition, Mechanism, Prognosis

## Abstract

上皮细胞-间叶细胞转化（epithelial to mesenchymal transition, EMT）不仅在胚胎的发育过程中起着十分重要的作用，还参与非小细胞肺癌（non-small cell lung cancer, NSCLC）的转移过程。近期的研究发现，发生EMT的细胞不仅出现了形态的改变，还出现了相关表型的改变。既往有关EMT发生机制的研究多数是针对其他肿瘤的，因此很有必要研究NSCLC中是否有类似发生机制。随着研究的进展，EMT相关的基础研究逐渐被用于预测NSCLC的预后。本文将对NSCLC中EMT的发生机制及其临床应用的研究进展进行探讨。

## Abstract (en)

Epithelial to mesenchymal transition (EMT) has an important role in the development of embryo, as well as that in the metastasis of non-small cell lung cancer (NSCLC). Recent researches have demonstrated that both morphological and phenotypic conversions emerge in cells undergoing EMT. As most of relevant studies were on other cancers, it is essential to uncover whether it is the similar mechanisms accounting for EMT in NSCLC. With the progress of the studies, EMT-related basic researches are gradually applied to predicting the prognosis of NSCLC. The aim of this article was to discuss the mechanisms related to EMT emerging in NSCLC.

肺癌在男性的肿瘤相关性死亡中占首位，在女性的肿瘤相关性死亡中占第二位，目前已成为世界范围内肿瘤相关性死亡的主要原因之一^[1]^。非小细胞肺癌（non-small cell lung cancer, NSCLC）占肺癌的85%，其中肺腺癌在NSCLC中所占的比例呈逐年增长的趋势。尽管近些年来诊断和治疗手段得到了不断的改进，但是NSCLC患者的5年生存率（约15%）却未得到明显提高。转移是NSCLC患者的主要死因之一。但是，肿瘤细胞的具体转移机制仍然不是十分明确^[2]^。近期研究^[3-6]^发现，NSCLC等恶性肿瘤的转移与上皮细胞-间叶细胞转化（epithelial to mesenchymal transition, EMT）的发生密切相关。

## 1 EMT的定义与鉴别

传统的研究是从形态学上区分上皮细胞和间叶细胞^[7]^，因此，EMT往往被简单定义为从形态规则的上皮细胞向形态不规则的间叶细胞的转变。典型的肺上皮细胞彼此之间连接紧密，排列也十分规则^[8]^。上皮细胞还具有顶面和基底面之分，这种特征被定义为上皮细胞的“极性”。上皮细胞的两种不同表面与不同的基质相连。上皮细胞间的这种排列方式能够阻止上皮细胞从组织中脱离。肺的间叶细胞恰恰相反^[9]^。间叶细胞间的连接十分不紧密，排列方式也十分不规则。间叶细胞的外形十分瘦长，这种形态有利于间叶细胞的运动。因此，间叶细胞的运动比上皮细胞活跃，二者的运动方式十分不同。

分子生物学研究发现EMT发生的过程不仅是细胞形态的改变，还伴随着相关分子标记物的改变。例如，E-cadherin仅在上皮细胞中表达，而不在间叶细胞中表达^[10]^。因此，Hay^[11]^将EMT定义为细胞逐步丧失上皮细胞标志物，同时逐步获得间叶细胞标志物的过程。其中，上皮细胞标志物包括E-钙粘蛋白（E-cadherin）、斑珠蛋白（plakoglobin）、细胞角蛋白（cytokeratins）等，而间叶细胞标志物包括N-钙粘蛋白（N-cadherin）、波形蛋白（vimentin）、α平滑肌肌动蛋白（α smooth muscle actin, α-SMA）等。因此，应该至少从形态学和分子生物学两个方面鉴别某种肿瘤是否发生了EMT。

## 2 EMT的发生机制

肿瘤细胞发生EMT需要一定的诱因，而引起EMT发生的诱导因子可分为内源性的和外源性的。其中，内源性诱因包括某些基因的突变或过表达。突变后能够导致EMT发生的基因包括*p53*等^[12]^；过表达后能够导致EMT发生的基因包括*K*-*ras*、*c*-*myc*和*RGC32*等^[13-15]^。这些基因发生异常后不但能够使细胞的形态发生改变^[13]^，而且还能够下调上皮细胞标志物的表达^[12]^，同时上调间叶细胞标志物的表达^[14, 15]^。外源性诱因包括吸烟、乏氧诱导因子（hypoxia inducible factor, HIF）、转化生长因子-β（transforming growth factor β, TGF-β）等多种因素^[16-18]^。其中，吸烟能够通过上调淋巴样增强因子1（lymphoid enhancer factor 1, LEF1）和转录因子Slug，募集组蛋白去乙酰化酶（histone deacetylase, HDAC），进而抑制E-cadherin的表达，促进EMT的发生^[16]^。HIF-2α能够在mRNA水平上调转录因子Snail、Zeb1以及间叶细胞标记物vimentin的表达，促进EMT的发生^[17]^。TGF-β能够通过Smad依赖性信号通路^[19]^或非Smad依赖性信号通路^[20]^抑制上皮细胞相关标记物E-cadherin等的表达，同时促进间叶细胞相关标记物N-cadhein等的表达。

如上所述，某些转录因子也参与了EMT的发生。与EMT相关的转录抑制因子大致可分为两类：一类能够直接抑制E-cadherin的转录和活性，如Snail1、Snail2和E47等^[21]^；另一类间接抑制E-cadherin的转录，如Twist等^[22]^。值得注意的是，Snail可以通过抑制清道夫受体A5（scavenger receptor class A member 5, SCARA5）的表达促进NSCLC发生EMT，这一机制与其他肿瘤不太相同^[23]^。目前，Snail是否必须受上游信号的调节才能够激活EMT尚无定论，仍然需要进一步研究。

NSCLC发生EMT不仅仅与诱导因子和转录因子有关，还与某些信号通路密切相关。Hedgehog通路是在研究果蝇胚胎时首次被发现的，与细胞的生长、侵袭密切相关，对NSCLC细胞的存活十分关键^[24, 25]^。这一通路中关键的效应分子包括Gli转录因子等锌指蛋白。Gli1能够迅速促进Snail的转录，进而调节E-cadherin的表达，促进NSCLC发生EMT^[25]^。Mizuarai等^[26]^研究发现核糖体蛋白S6激酶p70S6K2是Gli的下游效应分子。通过抑制p70S6K2可以抑制NSCLC细胞系中Hedgehog通路的激活。Maitah等^[27]^研究发现Hedgehog通路还可以与其他通路共同作用，比如通过上调配体Shh可以增强TGF-β对EMT的诱导作用。因此，Hedgehog通路既可以直接导致NSCLC发生EMT，也可以联合TGF-β等其他通路间接促进NSCLC发生EMT。并且，Hegehog通路促进NSCLC发生EMT的具体机制比既往的研究复杂得多，需要进一步深入的研究。

环氧化酶-2（cyclooxygenase 2, COX-2）是一种诱导酶，能够催化花生四烯酸和类花生酸前列腺素合成，具有抗凋亡、促增殖、促进EMT发生的作用，与NSCLC患者的预后密切相关^[28]^。前列腺素E2（prostaglandin E2, PGE2）是COX-2的一种代谢产物，能够促进免疫抑制、诱导肺癌中EMT的发生^[29]^。在COX-2/PGE2通路中，PGE2通过上调Zeb1和Snail进而促进EMT的发生^[30]^。基于以上理论，研究人员研发出了一些COX-2抑制剂（如阿利考西），希望借此能够治疗NSCLC^[31]^。但是近期的研究^[32]^发现，并非所有的COX-2抑制剂都能够逆转EMT。有些COX-2抑制剂，如塞来昔布（celecoxib）反而能够促进EMT的发生。因此，在研制和应用COX-2抑制剂时应当十分慎重。

与EMT相关的TGF-β通路可分为两种：一种需要转录因子Smad参与^[19]^，而另一种不需要Smad参与^[20]^。在Smad依赖性TGF-β通路中，TGF-β首先与受体结合，激活的TGF-β受体Ⅰ促使转录因子Smad2和Smad3发生磷酸化，发生磷酸化的Smad2、Smad3与Smad4结合形成三聚物^[19]^。这种三聚物再被转入胞核内，与其他转录因子一起调节TGF-β靶基因（如*E*-*cadherin*基因）的表达^[19]^。但是，Chen等^[20]^发现TGF-β诱导NSCLC发生EMT往往是通过PI3K/Akt通路和MEK/Erk1/2通路等不依赖Smad的通路进行的。

此外，核转录因子κB（nuclear factor-κB, NF-κB）通路、Notch通路和Wnt通路也能促进EMT的发生。但是，多项研究^[33-37]^表明这些通路主要促进乳腺癌等其他肿瘤发生EMT。在NF-κB通路中，NF-κB能够通过激活Twist和Snail促进EMT的发生^[34, 38]^。有研究^[33]^还发现，包括Ras在内的许多EMT相关基因的激活是通过NF-κB通路进行的。这说明NF-κB通路可能远比目前的研究结果复杂，仍需要更进一步研究。在Notch通路中，转录因子Jagged1和Notch1的过表达可以诱导转录因子Slug的表达，而Slug通过抑制E-cadherin的表达、激活β-catenin以及抵抗失巢凋亡来促进EMT的发生^[39]^。Sahlgren等^[35]^的研究发现，Notch能够诱导HIF-1α向赖氨酰氧化酶（lysyl oxidase, LOX）聚集，进而增强乏氧介导的LOX上调，而LOX具有稳定Snail（Slug）蛋白的作用。因此，他们认为Notch通路可以通过介导乏氧刺激EMT的发生^[35]^。在Wnt通路中，Wnt蛋白能够与Frizzled受体结合，进而在Frizzled受体、脂蛋白受体相关性蛋白（lipoprotein receptor related protein, LRP）、Dishevelled分子和轴抑制蛋白（Axis inhibition protein, Axin）之间形成一种稳定的受体复合物，使Dishevelled分子发生磷酸化^[36]^。磷酸化的Dishevelled分子会抑制GSK-3β活性，而GSK-3β又具有促使转录因子Snail1发生磷酸化的作用^[37]^。这样，Snail1就会在细胞中不断积累，最终促使EMT的发生^[37]^。目前仍需要大量研究证实NSCLC是否也能够通过这些通路发生EMT。

## 3 EMT与miRNA

miRNA（或microRNA）是一种长度为20个-22个核苷酸的长链非编码RNA（long noncoding RNA），能够在转录后调节基因的表达^[40]^。在EMT相关的miRNA中，miR-200被研究得最多^[41]^。miRNA-200能够下调Zeb1 mRNA和Zeb2 mRNA的水平，上调E-cadherin水平^[42]^，抑制EMT的发生，进而抑制NSCLC的转移^[43]^。最近的研究^[44]^发现，MiR-134/487b/655能够通过抑制膜相关性鸟苷酸激酶的活性进而抑制肺腺癌中EMT的发生，减少肺腺癌对吉非替尼的耐药性。而miR-30a能够下调Snail的表达进而影响NSCLC细胞中E-cadherin和N-cadherin的表达水平^[45]^。但是，Kong等^[46]^发现，TGF-β在诱导小鼠乳腺上皮细胞发生EMT的同时还能够促进miR-155的表达。降低细胞中miR-155水平反而能够抑制EMT的发生、抑制细胞间紧密连接的降解，进而抑制细胞的侵袭性。反之，增加miR-155水平能够促进EMT的发生，增强细胞的侵袭性。NSCLC中是否也存在这种现象仍然不十分清楚。这说明miRNA调节EMT发生的机制不尽相同。

既然miRNA能够抑制EMT而EMT又与NSCLC的转移相关，那么miRNA能否有效地预测NSCLC的预后呢？Gao等^[47]^发现miR-21的高表达（HR=5.993, 95%CI: 2.518-14.264; *P*＜0.001）、miR-181a的低表达（HR=0.328, 95%CI: 0.142-0.756; *P*=0.009）与患者的总生存期密切相关，而与TNM分期和淋巴结转移情况无关。Li^[48]^的研究也发现miR-146的高表达与NSCLC患者的预后密切相关，而与TNM分期和淋巴结转移情况无关。可见，miRNA与NSCLC的总生存期相关，而其预后价值仍需要更深入的研究。

## 4 EMT与肿瘤干细胞

肿瘤干细胞（cancer stem cells, CSC）是能够自我更新并能够分化成多种肿瘤细胞系的一类细胞。肿瘤干细胞理论最初只是一种设想，直到1997年首次通过实验证明肿瘤干细胞的存在^[49]^。Mani等^[50]^首次报道了EMT的发生能够导致肿瘤细胞成为肿瘤干细胞。他们发现经过球体培养的上皮细胞可以向干细胞样细胞转变，并且能够获得间叶细胞的某些表型^[50]^。Pirozzi等^[51]^发现，经过TGF-β1处理的NSCLC细胞系不但发生了EMT，还能够表达肿瘤干细胞的某些分子标记物，如Oct4、Nanog、Sox2、c-kit和CD133等。因此，我们推测，所谓EMT或许就是上皮细胞向肿瘤干细胞转变的一个过程。如果这一猜想成立的话，那新的问题出现了：既然肿瘤干细胞是能够自我更新并能够分化成多种肿瘤细胞系的一类细胞，那肿瘤干细胞的分化是否也能够导致EMT的发生。这些猜想尚需要大量的研究证实。

## 5 EMT与NSCLC预后的相关性

目前，有关EMT与NSCLC预后的研究多数是从相关标记物的临床意义入手的。与EMT相关的分子标志物包括上皮细胞标志物以及间叶细胞标志物、相关转录因子等^[6, 11]^。

E-cadherin是上皮细胞特异性标志物，表达水平在EMT的发生过程中逐渐降低^[11]^。Sulzer等^[4]^发现E-cadherin表达情况与NSCLC患者的预后密切相关。111例接受手术的患者中，高表达E-cadherin的患者的3年生存率是60%，而低表达E-cadherin的患者的3年生存率仅32%。但是，Tarin^[52]^通过回顾文献及自己既往的研究后认为EMT在肿瘤的进展中并不一定具有意义。Prudkin等^[53]^发现：E-cadherin表达的减少和N-cadherin的过表达与肺癌的预后不相关，且几乎所有的肺癌标本都存在EMT表型的变化。他们还发现，与肺癌的原发灶相比，脑转移瘤能够表达更高水平的E-cadherin^[53]^。Wu等^[5]^对13项研究中的2, 274例患者进行了*meta*分析，结果发现E-cadherin的下降确实与NSCLC患者的预后相关，但是与Ⅰ期患者的预后无关。

因此，EMT相关标志物与NSCLC患者预后的具体关系需要进一步研究及更加深入的分析，而首先需要解决的问题是筛选更多可供肺癌研究借鉴的相关标记物。例如，N-cadherin和Vimentin是间叶细胞特异性标志物，其表达水平在EMT的发生过程中逐渐增加^[11]^，同样可以作为检测EMT发生的特异性指标。Luo等^[54, 55]^发现胞核中N-cadherin和Vimentin的阳性表达率与鼻咽癌患者的临床分期相关（*P*＜0.001）。既然如此，N-cadherin和Vimentin与NSCLC的临床分期是否也存在一定的相关性呢？Hui等^[6]^发现Ⅲ-Ⅳ期与Ⅰ期和Ⅱ期NSCLC中N-cadherin的表达阳性率分别为39.58%、31.58%和23.53%（*P*＜0.01），而低、中、高分化NSCLC中N-cadherin表达阳性率分别为64.10%、35.71%和16.0%（*P*＜0.01）。Dauphin等^[56]^发现Vimentin的高表达与NSCLC的远处转移（*P*=0.024）和较晚分期（*P*=0.002, 8）相关。可见，N-cadherin和Vimentin的表达情况与NSCLC的分期同样存在一定的相关性。

转录因子在EMT的发生过程中有十分重要的调控作用，那么通过检测肿瘤细胞中转录因子的表达情况应该可以明确EMT的发生情况及其预后价值。Yin等^[57]^对107例骨肉瘤标本进行免疫组化染色，结果发现骨肉瘤中Twist的阳性表达率为31.8 %（34/107），并且Twist表达阳性的骨肉瘤患者在总生存期和无进展生存期方面均较差（*P*＜0.05）。类似地，Hui等^[6]^发现NSCLC中Twist的表达阳性率为38%，明显高于瘤旁正常组织（*P*＜0.01）。并且，Twist的表达情况与NSCLC的分期和分级相关。Ⅲ期-Ⅳ期NSCLC中Twist的表达率（56.25%）明显高于Ⅰ期（21.05%, *P*＜0.01）或Ⅱ期（32.35%, *P*＜0.01）。低分化癌与中分化和高分化癌中Twist的表达阳性率也各不相同，分别为66.67%、26.79%和20.00%（*P*＜0.01）。有淋巴结转移与无淋巴结转移的病例中Twist的表达情况也存在一定的差异（48.58% *vs* 24.0%, *P*＜0.01）。这说明，Twist对NSCLC同样具有预后意义。但是，Twist的过表达并不是NSCLC的独立预后因素，与N-cadherin的过表达呈一定的相关性（*R*=0.565, *P*＜0.001）^[6]^。

尽管上述的研究表明EMT相关的标记物与NSCLC患者的预后存在一定相关性，但这些研究仅仅是从分子生物学方面对EMT进行研究。既然EMT的鉴别至少需要从形态学和分子生物学两个方面进行，那么研究人员也应该将形态学因素考虑在内。

## 6 问题与展望

目前，有关EMT的研究虽然取得了一定的成果，但仍然面临许多问题。首先，多数研究表明EMT相关标志物与肿瘤的转移呈一定的相关性，但这些研究并未证明EMT与肿瘤进展是否真的存在一定的顺序关系或因果关系。Chui^[58]^认为，累积的遗传学和表观遗传学改变以及肿瘤微环境才是导致肿瘤获得侵袭性的重要因素，而EMT并非肿瘤发生远处转移的必要步骤。可见，EMT与肿瘤进展的关系尚存在一定的争论，仍需要深入的研究。其次，EMT相关基础研究应该不断地应用于临床。例如，Brachyury是最近发现的一种T-box转录因子，能够在多种肿瘤中表达^[59]^。Brachyury的高表达能够诱导上皮细胞发生EMT。针对这种转录因子，研究人员研发出了一种肿瘤疫苗，希望能够用这种疫苗治疗晚期NSCLC^[60]^。因此，在解决相关争论的同时，不断将EMT与NSCLC的免疫治疗相结合或许是未来的研究方向之一。
